# Toward a Structural Health Monitoring Methodology for Concrete Structures under Dynamic Loads Using Embedded FBG Sensors and Strain Mapping Techniques

**DOI:** 10.3390/s22124569

**Published:** 2022-06-17

**Authors:** Alejandra Amaya, Julián Sierra-Pérez

**Affiliations:** Grupo de Investigación en Ingeniería Aeroespacial, Escuela de Ingenierias, Universidad Pontificia Bolivariana, Sede Central Medellín, Circular 1 70-01, Medellín 050031, Colombia; alejandra.amaya@upb.edu.co

**Keywords:** structural health monitoring (SHM), fiber optic sensors (FOSs), fiber Bragg gratings (FBGs), pattern recognition, reinforced concrete structures, data driven

## Abstract

A data-driven-based methodology for SHM in reinforced concrete structures using embedded fiber optic sensors and pattern recognition techniques is presented. A prototype of a reinforced concrete structure was built and instrumented in a novel fashion with FBGs bonded directly to the reinforcing steel bars, which, in turn, were embedded into the concrete structure. The structure was dynamically loaded using a shaker. Superficial positive damages were induced using bonded thin steel plates. Data for pristine and damaged states were acquired. Classifiers based on Mahalanobis’ distance of the covariance data matrix were developed for both supervised and unsupervised pattern recognition with an accuracy of up to 98%. It was demonstrated that the proposed sensing scheme in conjunction with the developed supervised and unsupervised pattern recognition techniques allows the detection of slight stiffness changes promoted by damages, even when strains are very small and the changes of these associated with the damage occurrence may seem negligible.

## 1. Introduction

Civil structures during their useful life are exposed to different conditions that can affect their correct performance, such as weather conditions, high operating temperatures, seismic activities, overloads, etc., which can cause early deterioration. These circumstances have aroused interest around the world in the development of methodologies and techniques to detect unforeseen damage that can put human lives at risk [1,2].

Structures designed with reinforced concrete can undergo, throughout their useful life, deterioration caused by multiple reasons; one of these is the increase of loads in service. These load increases can be caused by unpredictable forces of nature, such as earthquakes.

There are several methodologies to evaluate the behavior of structures and generate alerts to prevent failures. Some of these methodologies are structural health monitoring (SHM), condition monitoring (CM), nondestructive testing (NDT), health and usage monitoring systems (HUMSs), and damage prognosis (DP), among others [3].

SHM presents significant advantages over other methodologies, some of which are: its ability to periodically update the state of the structure and provide information necessary to determine the ability of the structure to continue in operation. The SHM can be used to determine in real-time the state of the structure under a non-predictable condition such as a telluric movement [3] and can use high-tech devices such as fiber optic sensors [4]. Additionally, SHM implements pattern recognition techniques through machine learning algorithms as an integral part of the damage detection, classification, and assessment process [3].

The implementation of SHM in the civil industry has addressed cases such as monitoring of dams [5], buildings and bridges [6,7,8,9], offshore rigs [10], underwater tunnels [11], and timber structures [12], among many others.

SHM, as a methodology that implements information acquisition methods assisted by a wide variety of sensors, is directly affected by advances in sensing. Some of the sensors that have been implemented and are still being implemented are piezoelectric sensors, position sensors (GPS), fiber optic sensors, electrical strain gauges, accelerometers, inclinometers, etc. [13,14,15]. In the last decade, special attention has been given to fiber optic sensors due to their advantages and applicability in the field of SHM [16,17,18].

The remarkable properties presented by fiber optic sensors (FOSs), in particular fiber Bragg gratings (FBGs) (one kind of FOS), have evidenced their ability to be used in structural monitoring systems in civil structures. FOSs are immune to electromagnetic interference, are lightweight, their manufacturing process allows locating several sensors in a single fiber (multiplexing), and they present high fatigue resistance, high sensitivity, and present minimum invasion, which allows embedding the sensors in structures. These advantages make them suitable for applications in smart structures and SHM in civil structures [19,20,21,22,23,24,25].

There are several examples of the implementation of FBGs for the measurement of strains in concrete structures at the laboratory level [26,27,28,29,30,31,32]. However, and despite all its advantages, the implementation in real concrete structures of embedded FOSs is common. Perhaps one of the most representative works reported in the literature is a compilation of experimental studies on fiber optic sensors embedded in concrete [33].

However, under the framework of the prediction and prevention of structural damage in civil structures, a large number of methodologies in conjunction with several advanced sensing techniques have been developed and tested; however, most of them require complex data acquisition and processing systems, long acquisition and evaluation times, robust energy sources, and high installation and maintenance costs.

The aforementioned idea shows the opportunity to develop a simple, robust, and low-cost methodology for structural health monitoring in the construction field, which allows performing tasks such as assistance in the construction process of civil structures, a subsequent evaluation, and the determination of the performance of the structure under normal or abnormal operating conditions that may lead to structural failure.

## 2. Materials and Methods

### 2.1. Framed Structure Dimensioning

Initially, the design and building of the prototype of a simple framed structure were performed. The variables to be measured in the structure, the strategic measurement points, and the expected ranges of the measurements were identified.

The variables to be measured in the structure are strains produced under dynamic loads that resemble a low-to-medium-intensity telluric movement. The location of the measurement points coincides with the points where the greatest magnitude of theoretical strains occurs (midpoints and points of greatest deflection in both beams and columns).

It has been reported that, when strain mapping techniques are used, a signal-to-noise ratio (SNR) greater than 50 is desired to maximize the probability of damage detection since strain-based pattern recognition techniques work better when patterns for pristine condition and damaged conditions can be differentiated. As the strain magnitudes become larger and the SNR increases, differences between such patterns become more noticeable [34,35]. In this experiment, a Micron Optics SM130-700 fiber optic interrogator was used with an approximate accuracy of 1.25 pm (which means ±1 µε for strain measurements and ±0.125 °C for temperature measurements) for the used FBGs; therefore, strains in the structure over ±50 µε were desired; however, only maximum levels of 30 µε were reached without this, implying that the methodology does not work correctly as demonstrated in the present study.

**Figure 1:**
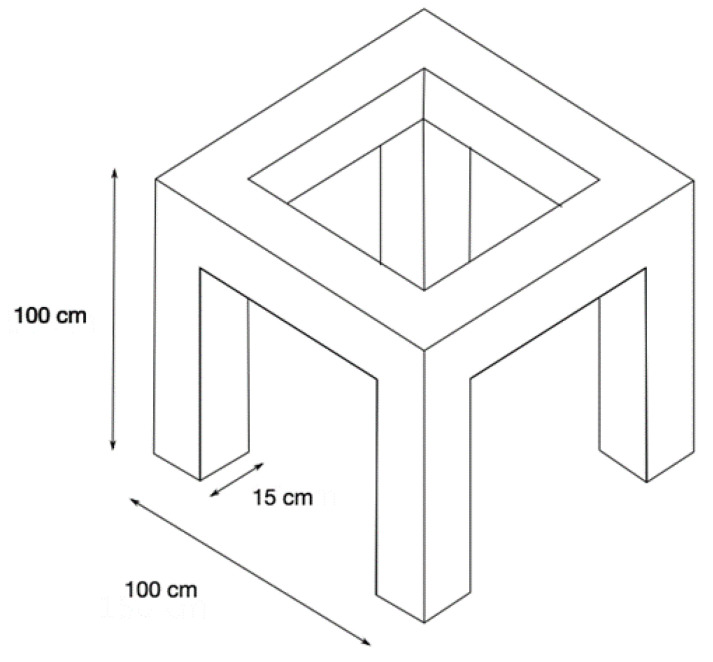
General dimensions of the framed structure.

Bearing in mind the above considerations, the design of the framed structure shown in Figure 1 was performed. This structure has a height of 100 cm, a width of 100 cm, and a length of 100 cm; each of its beams and columns has a cross-section of 15 × 15 cm. A homogeneous cross-section was selected so that the structure had the same inertia (same stiffness) in both axes of the section.

The prototype of the framed structure was built with corrugated steel reinforcements of 6.35 mm in diameter, using conventional concrete, which has a compressive strength of around 21 MPa.

The framed structure was designed using the buckling calculation method for columns and the analysis method for reinforced concrete beams presented by Hibbeler [36].

### 2.2. Sensing Scheme Design

Once the framed structure was dimensioned, two different configurations for FBG sensors were defined, one for beams and one for columns. The FBG sensors selected to be embedded into the structure have a reflection greater than 90%, a full-width at half-maximum (FWHM) less than 0.3 nm, grating lengths of 10 mm, and wavelengths in a range between 1510 and 1590 nm.

For each column, two fiber optic lines were designed with a total length of 115 cm, each one having two FBGs. In each fiber optic line, one of the sensors was intended for strain measurement and the other one for temperature measurement to perform a thermal compensation. For each beam, also two fiber optic lines were designed with a length equal to that of the columns, except that each fiber optic line has four FBGs. One FBG is intended for temperature measurement (thermal compensation), and the other three are for strain measurements. It is important to notice that due the short dimensions of the framed structure and the short distance between strain sensors, it was decided to use only one temperature sensor for thermal compensation in each one of the beams. The supposition is that the temperature in such a short length is approximately homogeneous during the experiments. In a bigger structure, it would be wise to use independent temperature sensors for each strain sensor to guarantee a strain measurement free of thermal effects.

**Figure 2:**
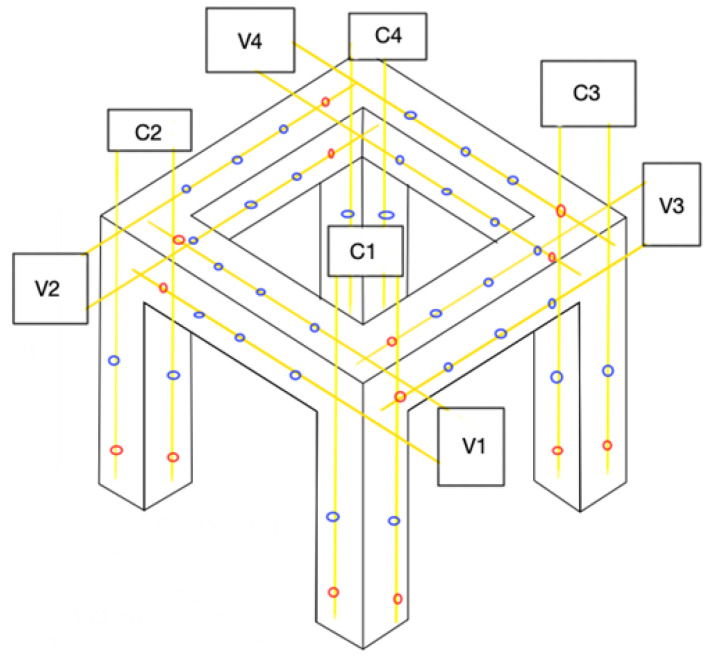
Schematic location of fiber optic lines with sensors. Blue dots represent strain sensors and red dots temperature sensors.

Figure 2 shows a schematic of the location of the fiber optic lines for the whole framed structure (beams and columns). As can be seen, there are a total of 16 fiber optic lines and a total of 32 FBGs for strain measurements and 16 for temperature measurements.

**Figure 3:**
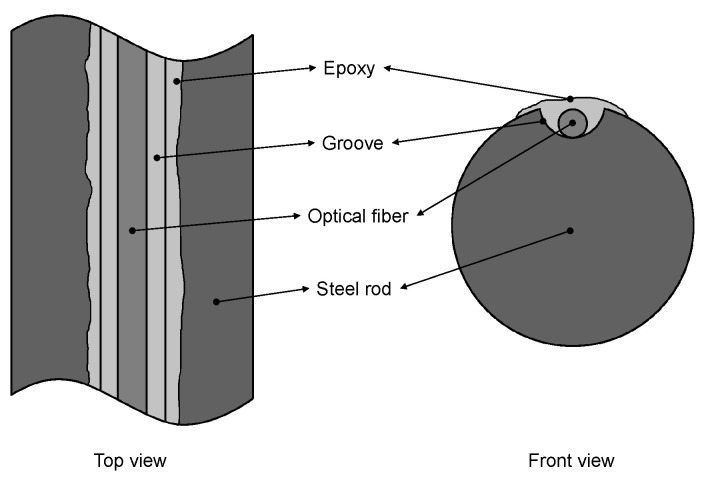
Schematic view of reinforcing steel bars instrumented with FBGs.

The FBGs were bonded using cyanoacrylate into 1 mm-wide and 1 mm-deep grooves made in each one of the reinforcing steel bars by CNC milling. Epoxy resin was used to cover and protect the optical fiber inside the grooves in a subsequent step. Flexible tubes were used to route the pigtails to the projection boxes where the optical couplers were installed. In Figure 3, a schematic view of reinforcing steel bars instrumented with FBGs can be appreciated.

**Figure 4:**
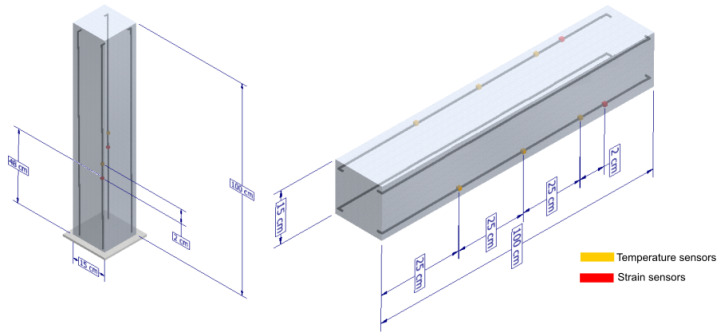
Detailed view of sensor location. **Left**: columns. **Right**: beams.

Figure 4 shows the detailed location of the fiber optic lines in beams and columns. As can be seen, there are a total of 12 strain sensors plus four temperature sensors in each beam and four strain sensors plus four temperature sensors in each column.

As shown in Figure 2, four fiber optic lines converge at each vertex of the structure, two from a column and two from a beam. Each of the four lines converging at each vertex has an FC/APC termination, which is connected to a 4:1 splitter. Finally, four 4:1 splitters were connected to the fiber optic interrogator (which has four channels) through four patch cords. In this way, the signals from 12 FBGs converge to a single channel of the fiber optic interrogator. It was ensured that each of the sensors had a different wavelength and had sufficient separation between them so that there was no overlapping of wavelengths during system operation.

Table 1 shows the FBGs’ wavelengths for the columns’ sensors, and Table 2 shows the FBGs’ wavelengths for beams’ sensors; additionally, the first column of both tables (Table 1 and Table 2) shows the nomenclature corresponding to the location of each sensor according to the diagram presented in Figure 2.

**Figure 5:**
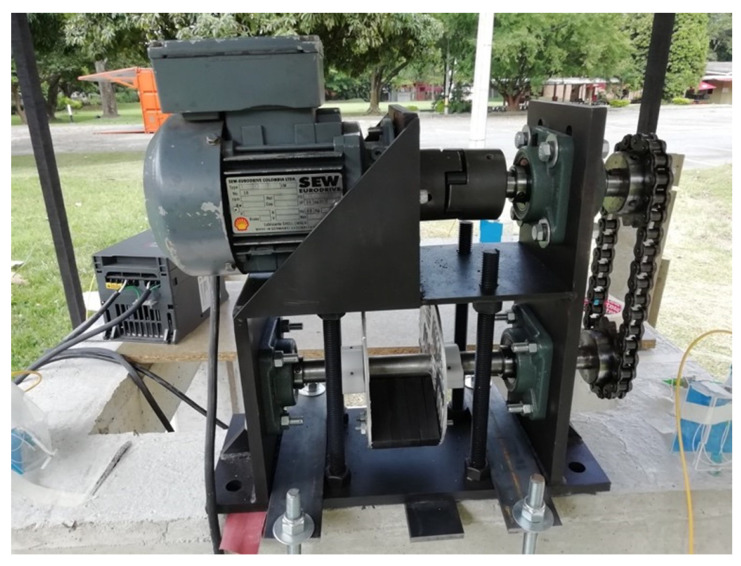
Mechanical shaker.

The behavior of the framed structure was evaluated versus different excitation frequencies each, with an associated force magnitude: 15 Hz with 808.9 N, 16.2 Hz with 943.4 N, 17.5 Hz with 1100.9 N, and 20 Hz with 1438 N. Each excitation frequency was used to evaluate two structural states: undamaged and damaged. The excitation was generated through a shaker, which serves to simulate low-to-medium-magnitude seismic activity. The mechanical shaker consisted of a rotating eccentric mass, allowing the generation of periodic excitation in the structure at different frequencies. Figure 5 shows the mechanical shaker composed of masses attached to a shaft, which is connected through a sprocket–chain transmission to a Sew-Eurodrive electric motor with a power of 0.37 KW at 30 rpm.

The “undamaged” state refers to the response of the structure in pristine conditions. The “damaged” state refers to the response of the structure with the presence of some artificially induced alteration or disturbance (damages). In the present study, the artificial positive damages consisted of 15 × 15 cm by 3 mm thick steel sheets. The artificial damages were adhered to the structure using the following methodology: first, the surfaces to adhere (both the structure and the steel sheet) were prepared by polishing these with 80 grit sandpaper and, then, with a metal bristle brush to generate deep scratches that would allow greater adhesion between them; then, the surfaces were cleaned with acetone and the sheets adhered to the structure with Loctite bi-component high-strength epoxy. These induced artificial damages increase the local and global stiffness of the structure by locally producing a change in the stiffness of the material and the inertia of the cross-section.

**Figure 6:**
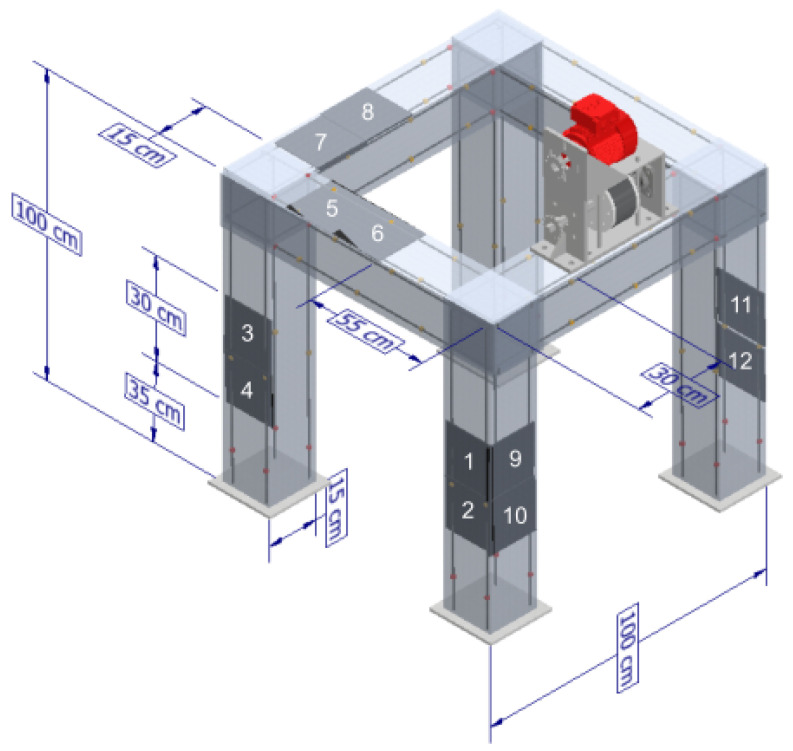
Location of artificial positive damages. Yellow dots represent strain sensors and red dots temperature sensors.

The location of the positive artificial damages was carried out in such a way that the change in the stiffness of the structure is greater in the most critical areas of the structure, i.e., areas of maximum deflection in beams and columns. Figure 6 shows the location of the damages. Additionally, the plates were numbered to define different levels of damage in the structure. The structure with all steel plates attached represented the most severe damage (highest induced stiffness), that is, Damage 5. Progressively, sheets were peeled off, decreasing the severity of the damage. Damage 4 corresponds to steel plates from 1 to 10, Damage 3 corresponds to steel plates from 1 to 6, Damage 2 corresponds to steel plates from 1 to 4, and Damage 2 corresponds to steel plates 1 and 2, see Figure 6. In total, five levels of damage severity were used.

In this way, data were obtained for the pristine condition and for each damage severity level for each excitation frequency. Each measurement was made for 15 s. A total of 100 measurements were performed with a total duration of 1500 s.

**Figure 7:**
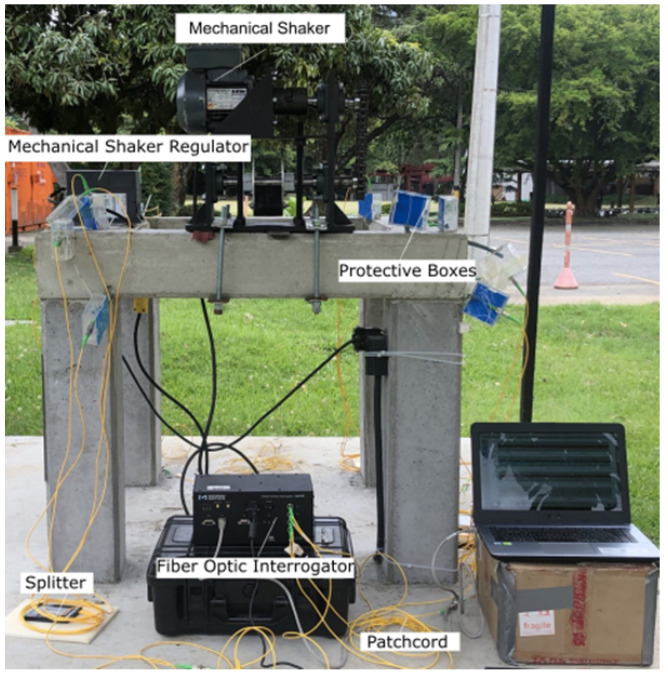
Experiment layout.

Figure 7 shows the final layout of the experiment, together with the location of the mechanical shaker on it.

## 3. Results and Discussion

The data obtained in each measurement were stored in $M_{ij}$ matrices where *i* is the number of rows and *j* is the number of columns. In this case, the rows correspond to the time, and the columns correspond to the different FBGs used.

**Figure 8:**
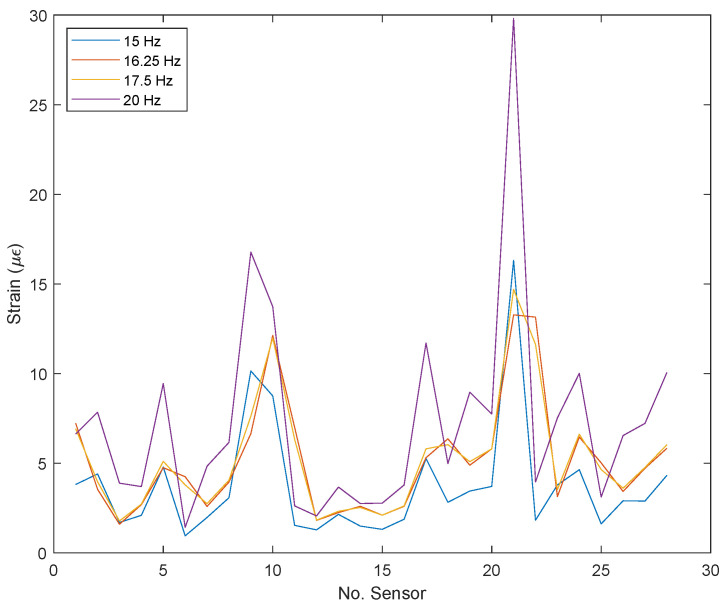
Strains for pristine condition versus sensor number (FBG number) for all excitation frequencies.

Due to an unforeseen event during the construction of the structure, the number of FBGs was reduced to 42, 28 strain sensors and 14 temperature sensors. Figure 8 shows the standardized average strain of the 28 strain FBGs for the undamaged condition, for each excitation frequency; in this way, it is possible to appreciate which sensors are more influenced by the stimulus generated by the mechanical shaker.

This was achieved by applying Equation (1) with a standard normalization $\left(\bar{X}\right)$, which, using its mean (μ) and standard deviation (σ), allows the data to be adjusted from their mean and distributed according to their standard deviation. This standardization was applied to the grouped data obtained during the experimental phase of the undamaged structure. Results are presented in Figure 8. Several predominant peaks are observed, which coincide with sensors numbered 2, 5, 9, 17, 21, 24, and 26; these sensors were the most influenced by the location of the stimulus generated by the mechanical shaker, which allows verifying that the behavior of the sensor network (strain pattern) depends on the site of the application of the external stimulus.

From this result, it is possible to appreciate that the dynamic response of the structure (strain patterns) for different excitation frequencies is quite different. This supposes an issue for the damage detection problem since the excitation frequency acts as a damage-sensitive feature (strain field sensitive feature). For this reason, in the present work, frequencies were treated as different “load conditions”. In a real-life application, the load conditions’ isolation would be possible by using an extra sensor (e.g., an accelerometer) or, even, by performing a fast Fourier transform of the strain spectrum. In this way, different models for different frequencies can be built; otherwise, including several frequencies (load conditions) in the model, this could be very general and lose sensitivity.

It can be observed that the magnitudes of the strains measured are higher for the excitation frequency of 20 Hz and are around 20 to 30 µε. These strain levels are lower than desired as mentioned before to achieve an SNR of at least 50; therefore, it is challenging to perform damage detection with such data quality, but still possible by using the proposed methodology.

**Figure 9:**
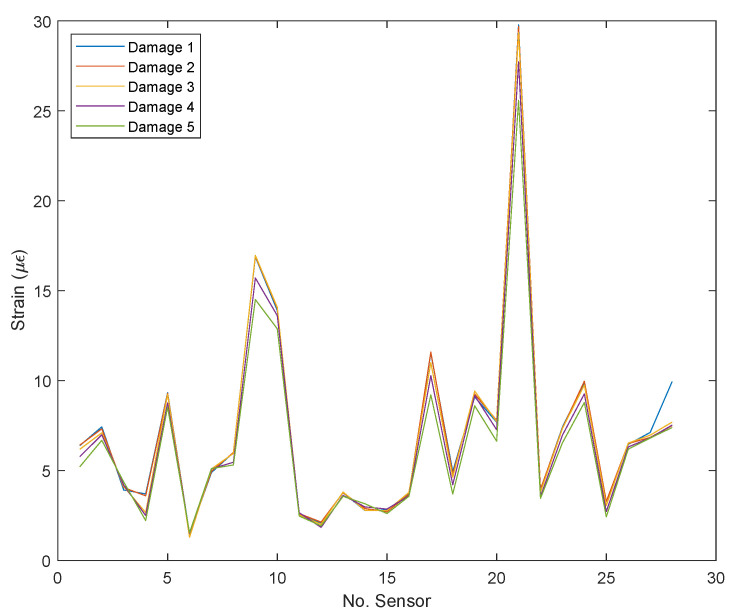
Average strain versus sensor number (FBG number) for an excitation frequency of 20 Hz and for the five levels of damage severity.

Figure 9 shows the standardized average strain for each sensor calculated from the dynamic strain spectrum for the damaged condition at the excitation frequency of 20 Hz, which is the one that exhibits the highest strain levels, as previously exposed. This figure makes it possible to show the slight differences in “strain patterns” promoted by damage occurrence even when damages are located in different positions and have different severities. This serves as a probe of the second issue of the strain-based damage detection in civil structures under dynamic loads: the low sensitivity of “plain” strain measurements to damage occurrence, and also serves as a justification for the use of pattern recognition techniques.

As can be seen in Figure 9, very similar “patterns” to those exhibited in Figure 8 for the 20 Hz excitation frequency occur; that is, there exist very similar strain patterns for the undamaged and damaged states, independent of damage severity. However, it is possible to observe that the most severe damage in Figure 9 (damage 5) presents a lower value of a few microstrains concerning other damages (related to the amplitude of its standardized average maximums); this means that a slight redistribution of strains occurs in the whole structure due to slight local changes in stiffness induced by artificial damages. Moreover, in this figure, it is possible to appreciate the changes in both local and global stiffness (changes in local maximums and changes in the whole strain trend) of the structure using the embedded sensor network; nevertheless, the strain changes are quite small, and again, the need for statistic pattern recognition techniques is evident.

Due the aforementioned issues, the implementation of statistical pattern recognition techniques to automatically classify the two states of the structure (damaged and undamaged) was implemented. Two approaches were used to generate two possible methods for structural health monitoring in framed concrete structures: supervised and unsupervised pattern recognition.

First, the experimentally obtained data were analyzed to determine to what statistical distribution they fit. A standard normalization of the data, using Equation (1), was performed for all excitation frequencies and damage levels. Ideally, experimental data fit a Gaussian distribution; for this reason, the normality test called the “Lilliefors test” was performed. The Lilliefors test mathematically determines the level of normality of a data set; it is used to test the null hypothesis that the data can be fit to a normal distribution using the difference between an empirical distribution function and a cumulative distribution function based on the data (see Equation (2)). If this hypothesis is tested, the distribution of the data can be fit, with a certain level of significance, to a normal distribution [37].

(1) $$\bar{X}=\left(X-\mu \right)/\sigma .$$

where μ is the sample mean and σ the sample standard deviation.

(2) D=sup|F(x)−G(x)|.

where *D* is the supremum, over all *X*, of the absolute value of the difference F(x)−G(x), F(x) is the cumulative distribution function of a normal distribution with mean zero and standard deviation one, and G(x) is the empirical distribution function of the values of $\bar{X}$.

The Lilliefors test provides a value called the *p*-value, which presents in a scalar value the validity of the acceptance or rejection of the null hypothesis of the normality test. When the test is performed, it is verified that the data are consistent with a normal distribution; this acceptance is due to the *p*-value obtained, which was 0.001.

After the validation of the normal distribution of the data obtained, the two damage detection methods to be used with the already standardized data were carried out, that is the supervised method and the unsupervised method.

### 3.1. Damage-Sensitive Feature

The Mahalanobis distance from the mean vector determined by μ to the respective variances determined by σ was used as a damage-sensitive feature. The Mahalanobis distance arises from a density function F(x), which describes the shape that a data distribution can have, allowing representing the probability using areas delimited by such a function F(x) [38].

Density functions can acquire multiple forms, depending on the physical problem being described by the data. In the case of a multivariate normal density, they conform to Equation (3), where the relationship of random variables that may or may not be correlated in *d* dimensions and that may or may not be dependent on each other is added to the univariate normal density expression; this is achieved using the covariance matrix. Covariance can be defined as a measure that associates two random independent variables, thus being able to determine if their relationship is high enough or not [38].

(3) $$p\left(x\right)=\frac{1}{{\left(2\pi \right)}^{\frac{d}{2}}{\left|\Sigma \right|}^{\frac{1}{2}}}e^{\left[-\frac{1}{2}{\left(x-\mu \right)}^{T}\Sigma^{-1}\left(x-\mu \right)\right]}.$$

where *x* is the different FBGs (variables), μ is the value of statistical means, Σ is the covariance matrix of dimensions d×d, |Σ| is the determinant of the covariance matrix, and $\Sigma^{-1}$ is its inverse; the symbol *T* denotes the transpose.

From Equation (3), it is inferred that the locus of constant density points is a hyperellipsoid with a constant quadratic shape determined by ${\left(x-\mu \right)}^{T}\Sigma^{-1}\left(x-\mu \right)$. The axes of such a hyperellipsoid, both major and minor, are determined by the eigenvectors of the covariance matrix and the eigenvalues of the covariance matrix, respectively. Quantitatively, the distance between the mean vector and the dispersion of samples defined in a hyperellipsoid is called the Mahalanobis square distance, with its mathematical formulation determined by Equation (4), where $r^{2}$ is the Mahalanobis distance.

(4) $$r^{2}={\left(x-\mu \right)}^{T}\Sigma^{-1}\left(x-\mu \right)$$

The Mahalanobis distance is used to determine the mean vector *u* for both the data for the undamaged and damaged conditions, and subsequently, the distance between these two vectors is determined. This allows providing visual proof that the data are different for each tested condition.

**Figure 10:**
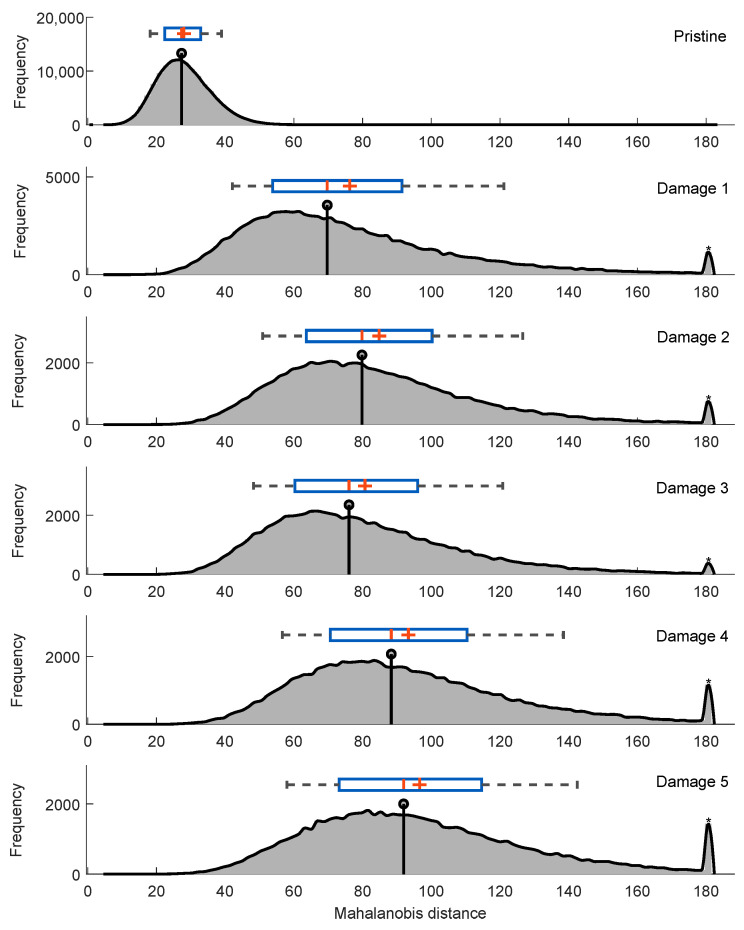
Mahalanobis distance vs. frequency for an excitation frequency of 20 Hz.

In a subsequent step, the estimated density function for each of the distances obtained is calculated. An example of the result for an excitation frequency of 20 Hz is presented in Figure 10. This figure shows a difference in distance between each of the density distributions (i.e., the distance between the behavior of the pristine structure under the 20 Hz frequency and each of the induced artificial damages); it is also observed that the most severe damage deviates in a more evident way from the behavior of the structure for the undamaged condition.

However, at the moment of establishing a limit where a boundary between undamaged and damaged is found, the existence of false positives and false negatives is found, i.e., values that are undamaged pass as if they were damaged and vice versa. For this reason, it is necessary to obtain a limit where it is possible to differentiate the states, which is called a decision threshold.

To solve the problem of how to determine the decision threshold with optimal performance for the proposed damage detection problem, two approaches were developed: the first one is the supervised learning method, and the second one is the unsupervised learning method [39].

### 3.2. Supervised Method

The receiver operating characteristic (ROC) method was used to define optimum decision thresholds. The ROC plots the probability of detection (POD) versus the probability of a false alarm (PFA) for a particular decision threshold and allows characterizing the performance of a model based on the discrimination between two classes [3]. Besides, the area under the curve (AUC) allows a “graphical view” of a model and facilitates the selection of an appropriate decision threshold [40]. In this way, this graphical method can be used to select an optimum threshold that allows fitting the data between two categories, maximizing metrics such as the accuracy, specificity, and sensitivity of the model [40].

In the experiment performed, there are only two classes, damaged and undamaged. Thus, a classifier (decision threshold) between both classes based on the ROC curve was obtained. This determines whether a set of samples belongs to one class or the other with the highest possible accuracy, sensitivity, and specificity.

**Figure 11:**
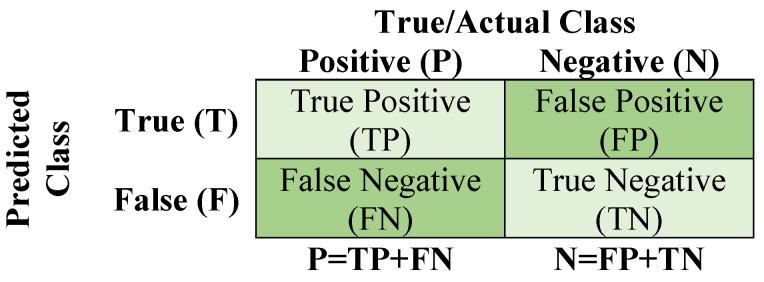
Confusion matrix for a binary problem.

The performance (accuracy, sensitivity, and specificity) of a classifier is related to the values in the confusion matrix. The confusion matrix contains the values for true positive (*TP*), true negative (*TN*), false positive (*FP*), and false negative (*FN*), as shown in Figure 11. *TP* indicates the samples correctly classified in the positive class (damaged), *TN* the samples correctly classified in the negative class (undamaged), *FP* the samples incorrectly classified as positive class (damaged), and *FN* the samples incorrectly classified as negative class (undamaged). An ideal classifier allows obtaining maximum *TP* and *TN* ratios while maintaining minimum *FP* and *FN* ratios.

The accuracy ($A_{cc}$) is a characteristic that determines the global performance of a classifier and is determined through Equation (5). The error rate (*ERR*) defines the error range in the classification in percentage and is determined by Equation (6). The sensitivity or the true positive rate (*TPR*) and specificity or the true negative rate (*TNR*) are representations of the balance between data correctly classified as positive or negative. These are represented by Equations (7) and (8), respectively.

(5) $$A_{cc}=\frac{TP+TN}{TP+TN+FP+FN}$$

(6) $$ERR=1-A_{cc}$$

(7) $$TPR=\frac{TP}{TP+FN}$$

(8) $$TNR=\frac{TN}{TN+FP}$$

**Figure 12:**
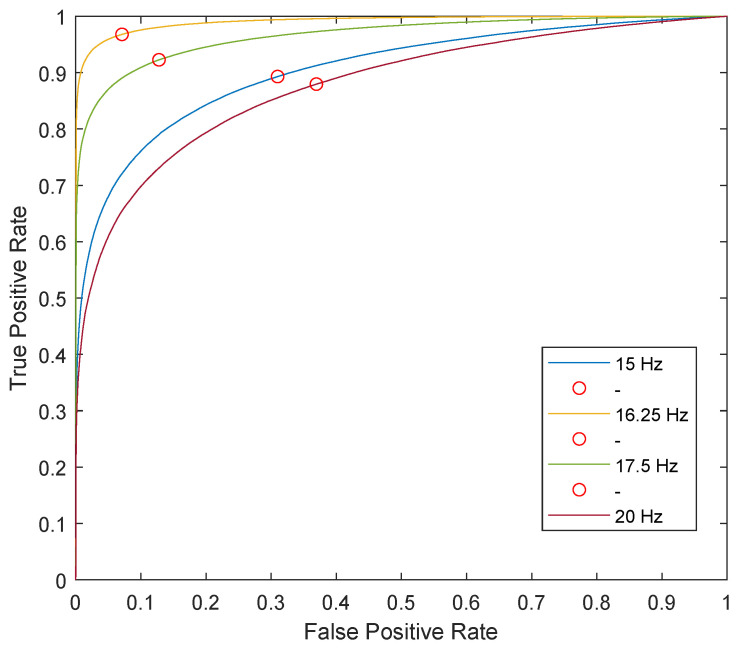
ROC curve for the Mahalanobis distance-based classification method.

In an ROC curve, the AUC is a metric parameter that takes values between zero and one and determines the success of the classifier. If the AUC is close to one, it means that it presents assertive results [40]. The ROC curves for four excitation frequencies can be observed in Figure 12. Red circles in Figure 12 represent the optimum decision threshold for each class.

Since the ROC curve represents all the decision thresholds that the binary classification problem can have, some of these decision thresholds are not optimal due to the percentage of performance they represent; therefore, optimum values are obtained using a “score”, and the optimal decision thresholds will depend on the score that presents the best balance between false positives and false negatives. The results for the optimum decision thresholds are presented in Table 3, as well as the related AUCs.

**Figure 13:**
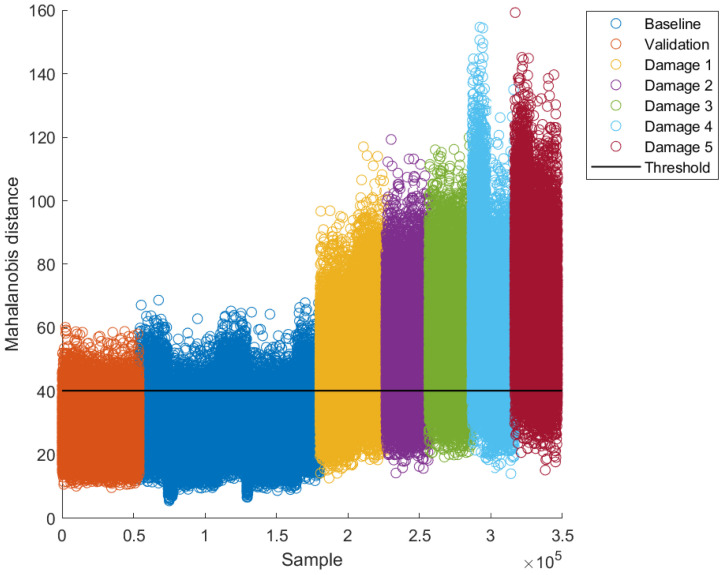
Results for an excitation frequency of 16.25 Hz with optimum threshold.

**Figure 14:**
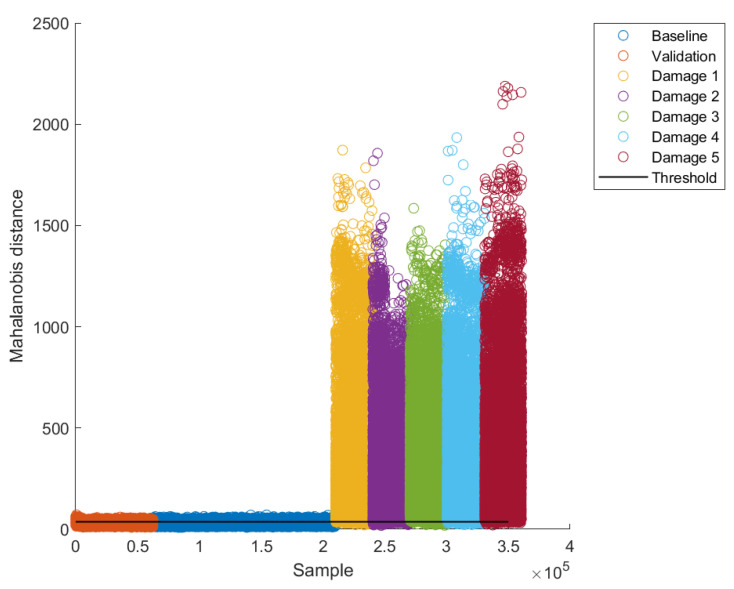
Results for an excitation frequency of 17.5 Hz with optimum threshold.

Once the thresholds were calculated, the baseline models for each excitation frequency were built using 70% of undamaged data (“Baseline” in Figure 13 and Figure 14). The remaining 30% of undamaged data were used to validate the model (“Validation” in Figure 13 and Figure 14). After building the baseline models, data for damaged cases were projected into their respective model and classified according to thresholds as damaged or undamaged; this is why such a threshold is commonly called a “damage threshold”. A couple of examples of such results for two excitation frequencies (16.25 Hz and 17.5 Hz, respectively) are presented in Figure 13 and Figure 14.

As can be seen, a large proportion of baseline data fall below the damage threshold for both excitation frequencies (Figure 13 and Figure 14). Moreover, also a large proportion of validation data fall below the damage threshold. On the other hand, most data for damaged cases fall above the damage threshold. This intuitively allows inferring that the damage detection methodology works properly. Through a quantitative analysis of methodology performance, such inference can be probed or denied. Table 4 presents the values of performance metrics for the Mahalanobis-distance-based classification method developed, by using the optimum decision thresholds selected.

As can be seen, the accuracy lies between 69% and 93%, the 20 Hz excitation frequency having the worst accuracy and 16.25 Hz the best accuracy.

As can be seen in Table 4, excitation frequencies of 16.25 and 17.5 Hz showed a better global performance ($A_{cc}$); this is because there is less overlapping between the Mahalanobis distances, i.e., the undamaged and damaged conditions are better separated.

### 3.3. Unsupervised Method

As an unsupervised method, the use of confidence intervals through Chebyshev’s theorem as decision thresholds is proposed. Confidence intervals are interpreted as a quantitative method of uncertainty estimation, which is used to establish limits. By generating a margin of error, it is possible to use these, according to a certain confidence level, to establish the probability that, from such a limit, some values belong to a group and the others do not belong to it. In this case, Chebyshev’s theorem establishes a relationship that can exist between the mean (μ) and the standard deviation (σ); this relationship comprises the probability that a variable takes a given value between *k* standard deviations from its mean, where *k* is a constant that can take any real value. Equation (9) states this theorem [38].

(9) $$P\left(\mu -k\sigma <\mu +k\sigma \right)\geq 1-\frac{1}{k^{2}}$$

Equation (9) provides the reliability percentages at which the probability that the variable *x* lies between the values of the interval determined by μ±σ. Thus, if k=2, there will be a probability of 75% that the variable *x* is among those values of the interval; if k=3, one would have the reliability of 88% [41]. This theorem is presented as valid for any statistical distribution and presents reliable results since the theorem is mathematically provable [38].

This unsupervised method allows for a more generalized model, compared to the supervised model previously proposed, and is based on a baseline model built from the pristine condition of the structure; thus, it is not necessary to involve damage data in the model construction. However, damaged data were used for validating the model, as was done with the supervised method.

The results of the lower and upper limits for the baseline model for each of the established excitation frequencies and with a confidence level of 75% and 88% are observed in Table 5; that is, in Chebyshev’s theorem, *k* takes the values of 2 and 3, respectively. These confidence levels are selected since they are those that present a medium level of confidence and an acceptable performance according to the problem set out in the present work.

**Figure 15:**
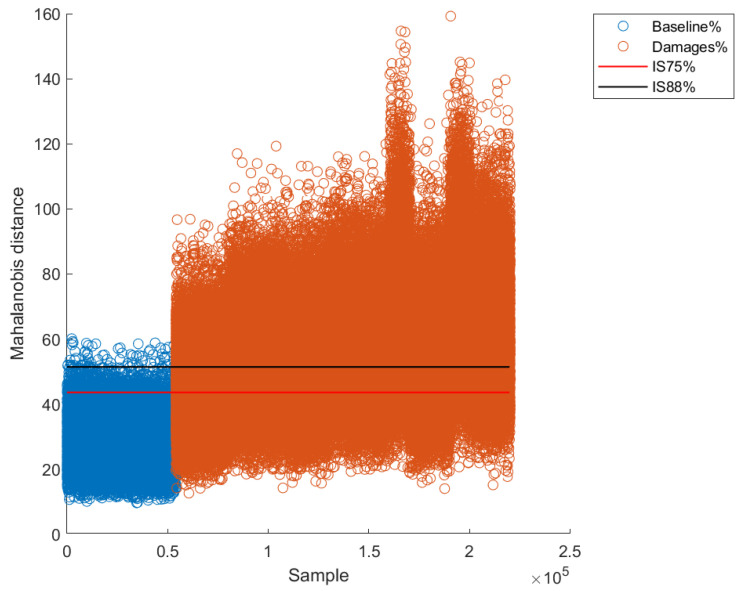
Confidence intervals for excitation frequency of 16.25 Hz.

**Figure 16:**
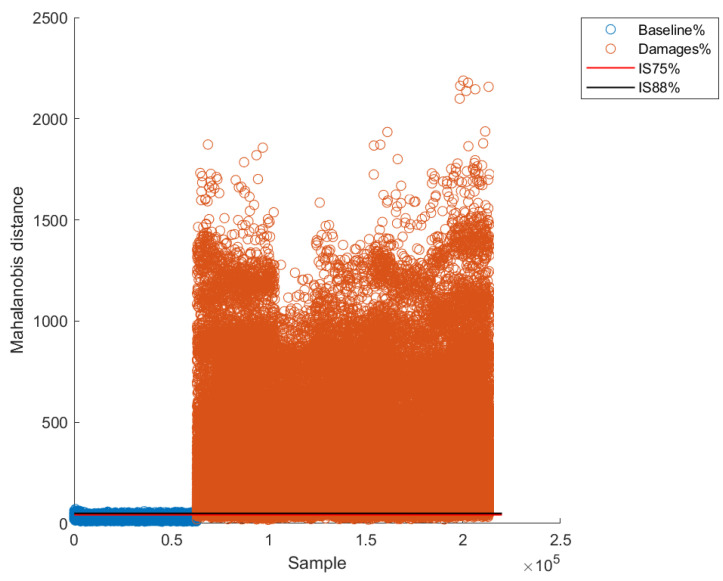
Confidence intervals for excitation frequency of 17.5 Hz.

After obtaining the intervals, the validation was carried out for each of the excitation frequencies. A couple of examples for two excitation frequencies are presented in Figure 15 and Figure 16. As can be observed, there is an observable difference between pristine Mahalanobis distances and damaged ones. However, and as expected, there are some excitation frequencies where the separation between pristine and damage is clearer. For instance, differences between pristine and damaged Mahalanobis distances are clearer for an excitation frequency of 15.5 Hz (Figure 16) than for an excitation frequency of 16.25 Hz (Figure 15).

Similar to the supervised method, quantitative metrics were used to evaluate the performance and effectiveness of the unsupervised method. Table 6 shows the metrics for the 75% confidence level and 88% confidence level. As can be observed, the accuracy for the worst result (for an excitation frequency of 20 Hz) fluctuates between 66% and 78% for 75% and 88% confidence intervals, respectively. This result is not surprising since it is possible that the structure enters into resonance at some frequencies, making less clear the separation between pristine and damaged patterns, causing a worst global performance. On the other hand, the best accuracy result was obtained for the excitation frequency of 17.5 Hz, being 98% for both confidence intervals (75% and 88%).

From the results obtained, it should be noted that the excitation frequency with the best performance was 17.5 Hz in both methods (supervised and unsupervised). In general, a good quantitative result of both methods was observed, validating the SHM methodology in a prototype of a reinforced concrete frame structure.

## 4. Conclusions

The use of embedded FBGs in a reinforced concrete structure was validated using the reinforcing steel bars as a carrier to protect the FBGs during the construction stage of the concrete structure. Load transfer between reinforcing steel bars, concrete, and superficial adhered positive damages was validated and demonstrated to be enough to detect such damages from the analysis of strain measurements performed at the reinforcing bars.

It was demonstrated that local superficial damage occurrence changes both the local and global structural stiffness; thus, it is possible to detect damage in a reinforced-concrete-framed structure by studying such stiffness changes.

It was concluded that the pattern recognition techniques used are, in terms of metrics, effective in detecting damage that produces local and global changes in the stiffness of the structure under dynamic loads, similar to those produced by medium-intensity telluric movements.

It was observed that the methodology proposed in this work is functional under low to medium excitation frequencies, presenting better performances at medium excitation frequencies. The use of the ROC curve as a supervised method of binary classification was validated, as well as Chebyshev’s confidence interval method as an unsupervised method.

## Figures and Tables

**Table 1:** Column sensor wavelengths (nm).

Nomenclature	Strain FBG No. 1	Temperature FBG
C11	1524.938	1529.909
C12	1515.071	1520.15
C21	1525.045	1530.077
C22	1515.171	1520.055
C31	1525.041	1530.001
C32	1515.135	1520.149
C41	1525.151	1529.891
C42	1514.799	1520.016

**Table 2:** Beam sensor wavelengths (nm).

Nomenclature	Strain FBG No. 1	Strain FBG No. 2	Strain FBG No. 3	Temperature FBG
V11	1570.071	1575.146	1580.007	1584.946
V12	1550.27	1555.05	1559.976	1565.047
V21	1569.977	1575.027	1579.967	1585.029
V22	1550.132	1555.295	1560.125	1565.018
V31	1569.809	1574.983	1579.996	1584.955
V32	1549.976	1555.086	1560.161	1565.041
V41	1569.832	1574.98	1579.855	1585.043
V42	1549.861	1554.861	1559.9	1565.019

**Table 3:** Optimal threshold values for each excitation frequency and its related AUC.

Excitation Frequency (Hz)	Threshold	AUC
15	30.72	0.9051
16.25	40.18	0.9912
17.5	36.33	0.9661
20	29.68	0.8774

**Table 4:** Methodology’s performance metrics.

Excitation Frequency (Hz)	$A_{\mathrm{cc}}$	ERR	TPR	TNR
15	0.74	0.25	0.89	0.69
16.25	0.93	0.06	0.96	0.92
17.5	0.88	0.11	0.92	0.87
20	0.69	0.30	0.87	0.63

**Table 5:** Intervals obtained by Chebyshev’s theorem.

Interval	Excitation Frequency (Hz)	75%	88%
Lower	15	15.1664	8.7497
Upper	15	40.8332	47.2499
Lower	16.25	12.3692	4.5539
Upper	16.25	43.6303	51.4456
Lower	17.5	13.5209	6.2814
Upper	17.5	42.4787	49.7182
Lower	20	14.9391	8.4088
Upper	20	41.0604	47.5908

**Table 6:** Performance metrics of the unsupervised method, 75% and 88% confidence intervals.

**75% Confidence Interval**				
**Excitation Frequency (Hz)**	$A_{cc}$	**ERR**	**TPR**	**TNR**
15	0.88	0.12	0.96	0.85
16.25	0.81	0.18	0.98	0.76
17.5	0.98	0.02	0.97	0.99
20	0.78	0.21	0.99	0.72
**88% Confidence Interval**				
**Excitation Frequency (Hz)**	$A_{cc}$	**ERR**	**TPR**	**TNR**
15	0.79	0.20	0.99	0.72
16.25	0.66	0.34	0.99	0.56
17.5	0.98	0.02	0.97	0.99
20	0.66	0.34	0.99	0.55
